# Transient Receptor Potential (TRP) Channels in Drug Discovery: Old Concepts & New Thoughts

**DOI:** 10.3390/ph10030064

**Published:** 2017-07-06

**Authors:** Susan Huang, Arpad Szallasi

**Affiliations:** 1 AbbVie Inc, 1 North Waukegan Road, North Chicago, IL 60064, USA; susan.huang@abbvie.com; 2 Baptist Medical Center, 800 Prudential Drive, Jacksonville, FL 32207, USA

2017 marks the 20th anniversary of the molecular cloning by David Julius and colleagues (1997) of the long sought-after capsaicin receptor, now known as TRPV1 (Transient Receptor Potential Vanilloid 1) [1]. This seminal discovery has opened up a “hot” new field of basic research and launched drug discovery efforts into the large family (by the latest count 28 mammalian members, 27 in humans) of TRP ion channels [2]. Indeed, it took less than a decade for the first potent, small molecule TRPV1 antagonists to enter phase 1 clinical trials [3]. Yet, despite the large amount of resources that has been invested in TRPV1 research, there are currently no TRPV1-targeted drugs in phase 3 clinical trials. In this special issue of Pharmaceuticals, we aim to capture the progress in the TRP channel field over the past twenty years, with 15 articles covering a variety of TRP channels and potential relevant disease states and applications.

Fitting to the root of TRP channel discovery, Mickle and colleagues provide a comprehensive review of the nociceptive TRP ion channels, including TRPV1 [4]. TRP channel activation by specific physical-chemical stimuli, expression in the nociceptive system, and involvement in various types of pain are discussed. They argue that inhibition of TRP channels expressed on nociceptive neurons represents a viable therapeutic pain target. Small molecule modulators of TRP channels that have already progressed into clinical development for the treatment of pain are also discussed.

Natural products offer a rich source of chemical diversity to identify novel drug candidates. Indeed, one in three new medicines approved by the FDA is derived from natural products. In sensory pharmacology, capsaicin (the pungent principle in hot chili peppers) and its ultrapotent analog, resiniferatoxin, are useful tools to dissect the pain pathway. Drs. Man-Kyo Chung and James Campbell summarize the sensory and physiological effects of capsaicin (a natural TRPV1 agonist) and the rationale of its therapeutic use [5]. They describe how the short-term excitatory effects of capsaicin (characterized by pungency or pain) are followed by a lasting refractory state in which the previously excited neurons are unresponsive to various unrelated stimuli. Mechanisms underlying capsaicin-induced analgesia both in the form of desensitization and denervation are discussed, along with potential clinical implications.

Dr. Dorothy Cimino-Brown continues the theme of TRPV1 agonism by discussing the therapeutic potential of the ultrapotent TRPV1 agonist, resiniferatoxin, as a “molecular scalpel” to achieve permanent pain relief [6]. The review recounts both preclinical studies (including bone cancer in companion dogs) and clinical trials in patients with advanced cancer pain. The pain relief and the improvement of quality of life observed in these limited studies warrant further exploration in more patients.

Capsaicin and resiniferatoxin are natural products. Existing TRPV1 antagonists have come from mass-screening of compound libraries. Drs. Carnevale and Rohacs suggest an alternative approach: rational, structure-based drug design [7]. They discuss the advances in elucidating the structure of TRPV1, and how these led to a better understanding of the mechanisms underlying agonist binding and channel function. Rational drug design based on structural information and computational modeling may generate libraries of compounds that hold promise as potent TRPV1 modulators.

TRPV3 is a cousin of TRPV1 [2]. Indeed, TRPV3 can form hetero-tetrameric channels with TRPV1, hence TRPV3 can reasonably be expected to potentially serve similar roles or impact TRPV1 function in some circumstances. Dr. Broad and colleagues review our current understanding of TRPV3 expression and function, as well as their potential clinical relevance [8]. In their paper, drug development efforts centered on TRPV3 by various pharmaceutical companies are summarized.

Exemplified by TRPV3, which is expressed in keratinocytes (where its gain-of-function mutation has been linked to Olmsted syndrome [9], a pruritic skin disorder), various TRP channels are found in different cell types in the skin. Drs. Caterina and Pang provide a comprehensive review of cutaneous TRP channel expression [10]. They summarize the growing body of evidence suggesting that these channels play an important role in skin physiology, from sensation through keratinocyte differentiation and barrier function to hair growth. Malfunction of these channels has been implicated in various disease states, including pruritus, dermatitis, hirsutism/alopecia, and cancer.

Unlike TRPV1 and TRPV3, that respond to heat, TRPM8 is a cold-activated channel expressed on nociceptive neurons. Drs. Weyer and Lehto discuss the on-going efforts to develop TRPM8 antagonists for the treatment of chronic pain and migraine [11]. The role of TRPM8 in mechanical and heat analgesia, cold hyperalgesia, bladder pain, and migraine is discussed. They note that TRPM8 appears to be analgesic in some cases but nociceptive in others—a challenging feature for the clinical development of TRPM8 antagonists.

Drs. Kumamoto and Fujita report the differential activation of TRPV1, TRPA1, and TRPM8 channels in rat spinal cord slices by stereoisomers using whole-cell patch clamp recording [12]. They show that carvacrol and thymol (from the essential oil of thyme), carvone (from caraway), and cineole (from eucalyptol) increase the frequency of spontaneous excitatory postsynaptic currents; however, these presynaptic activities differ when activated by different stereoisomers, suggesting potential additional consideration for modulating these channels.

Inhaled capsaicin has been used clinically to identify a subset of chronic cough patients. Dr. Eva Millqvist discusses the putative role of TRPV1 and TRPM8 in chronic idiopathic cough and cough hypersensitivity syndrome [13]. Indeed, chronic cough was a potential clinical indication for TRPV1 antagonism. Disappointingly, TRPV1 antagonists failed to show any antitussive effect in clinical trials. Thus, the role of TRPV1 in the pathogenesis of chronic cough remains speculative.

TRPA1 is another channel that is expressed in the respiratory tract. Mukhopadhyay and colleagues summarize the state of our understanding of the role of TRPA1 in chronic cough, asthma, chronic obstructive pulmonary disease, allergic rhinitis, and cystic fibrosis [14]. They describe the preclinical and clinical progress achieved so far with selected TRPA1 antagonists.

Another area of active TRP channel research is the area of cancer biology. Dr. Nelson Yee reviews the expression and role of TRPM7 in human malignancies, such as pancreatic, gastric, and breast carcinomas [15]. The potential of modulating TRPM7 as an anti-cancer therapy is discussed.

Drs. Grolez and Gkika focus on another member of the TRPM family, TRPM8, in prostate cancer [16]. They describe the androgen-dependent characteristics of TRMP8, and discuss the data implying a role for TRPM8 in prostate cancer cell proliferation, survival, and migration. They also note the promise of TRPM8 as a diagnostic marker in the clinic.

Drs. Zsombok and Derbenev discuss the emerging role of TRP channels in obesity and diabetes [17]. They review laboratory data on the regulation of hormonal release, energy expenditure, pancreatic function, and neurotransmitter release, and describe the effects of capsaicin in human subjects. Obesity is a world-wide epidemic. It is an attractive hypothesis that such an inexpensive and readily available dietary substance as capsaicin may be used for weight control.

Drs. Yamamoto and Shimizu review the role of another TRPM family member, TRPM2, in reactive oxygen species (ROS)-coupled diseases [18]. TRPM2 can be activated by ROS in an ADP-ribose-mediated manner to function as a transducer that converts oxidative stress into calcium signaling. The relevance to conditions such as inflammation, infection, ischemia-reperfusion, and Alzheimer’s disease is discussed.

Staying with the non-ligand mode of activation, Drs. Nagarajan and colleagues describe the ability of hydroxylation enzymes to regulate the channel activity of TRPA1 and TRPV3 [19]. The potential of targeting these enzymes as a means of modulating TRP channel activity is discussed.

The literature on TRP channels is immense. TRPV1 alone is a keyword in over 5000 publications searchable in PubMed. It is not possible to capture the entire literature in a single thematic issue. Consequently, the selection of articles presented in this issue represents a sampling of the literature, and is admittedly subjective. We tried to survey the wide range of human diseases in which TRP channels have been implicated, ranging from chronic pain through asthma and diabetes, to cancer, and highlight the channels that appear to hold the greatest promise for therapeutic targeting, in our opinion. Unfortunately, promising results obtained in laboratory species do not always yield equivalent results in clinical trials. Yet, from the richness of the investigations over the last 20 years, and the wide range of human conditions that have been implicated, it is fair to say that TRP channels constitute a formidable family of potential therapeutic targets that will likely continue to demand attention.
